# 

**Published:** 1882-09

**Authors:** 


### Pamphlets.

A study of rupture of the bladder, with some experiments bearing upon the closure of the vesical wound. By Alex. W. Stein, M.D , New York. Reprint from *Annals of Anatomy and Surgery.*

A review of Dr. Wooten's review of the causes that led to the death of the late Adj't Gen. John B. Jones. By Drs. Morris, McLaughlin and Swearingen, Austin, Texas.

The practice of gynæcology in ancient times. By Edward W. Jenks, M.D., LL.D., Chicago. Reprint from Gynecological Transactions, volume vi., 1882.

Letters and facts not heretofore published, touching the mental condition of Charles J. Guiteau since 1865, with the compliments of George M. Beard, M.D., New York.

Diphtheritic ulceration of the air-passages and its relation to pulmonary phthisis. By Jno. M. Mackenzie, M.D., Baltimore. Reprint from the Proceedings of the Medico-Chirurgical Faculty of Maryland.

Extensive ravages from lupus, with subsequent cicatrization, leaving but one small hole in the face, which represents both mouth and nose, and with complete closure of the anterior nasal orifices. By Julian J. Chisolm, M.D., Baltimore.

Report on ophthalmology, made to the Medico-Chirurgical Faculty of Maryland. By Julian J. Chisolm, M.D., Baltimore.

Class-work of the pupils of the Illinois asylum for feeble minded children, for the school year ending June 30, 1882, together with the commencement exercises held in the chapel of the asylum. C. T. Wilmur, M.D., superintendent, Lincoln, Ill.

Transactions of the Massachusetts Medico-Legal Society. Cambridge: Riverside Press. 1882.

Fourteenth annual report of the president of the Inebriates' Home, Fort Hamilton, N. Y., for the year 1881; also a statistical report of six hundred cases of alcoholic inebriety treated at the Inebriates' Home from November 1, 1879, to January 1, 1881. By Lewis D. Mason, M.D.

Treatment of consumption, indicated by the discoveries of Koch and others of its parasitic origin. By M. L. James, M.D., Richmond, Va. Reprint from *Virginia Medical Monthly.*

### INDEX TO VOLUME I.

Abdominal section for intestinal obstruction... 413
Abdominal tumor... 347
Absorption of sequestra... 690
Abscess in the abdominal walls... 662
A bogus medical college... 49[illegible]
Aceptic vaccine lymph... 537
Acne and sycosis, treatment of... 154
Acne, sulphur in... 248
Acne vulgaris... 419
Adjuvants, corrigents, etc... 238
Advance in therapeutics in 1880... 46
Air-passages, foreign bodies in... 747
"Allopathy"... 249
Alcohol in old age, beneficial effects of 211
Albumen, metaphosph ric test for... 93
Alcohol in therapeutics... 360
American Public Health Association.. 120
American Public Health Association, proceedings of... 212
American Medical Association, proceedings of... 583
A French court decision... 47
Alkaline water as a vehicle for the iodides and bromides... 102
Atresia of the vagina and uterus... 65
A new sanitary inspector... 491
Animal magnetism... 33
Artificial antiseptic tympanum... 155
A new vermifuge... 105
Aural cases, treatment of... 41
Aortic aneurism, electrolysis in... 548
Another drop in the bucket... 690
Anæsthesia, local, in neuralgia... 678
Augusta Health Board... 686
Analysis of milk... 689
Arsenic in the treatment of chorea... 606
Articular rheumatism, treatment of.. 303
A word for truth... 301
A plea for improved vaccination... 287
Antiseptic suture of the patella... 267
Antiseptic midwifery... 564
Antiseptics and drainage... 294
Asthma, grindelia robusta for... 534
Asthma... 301
Anæsthetics medico-legally considered... 555
Anæsthetics, danger of administering without witnesses... 369
Atlanta Medical Society, proceedings of...341, 400, 472, 528, 595, 662, 723
Bacteria of tuberculosis... 531
Bacteria in typhoid fever... 235
Bacillus of tuberculosis... 565
Badgering scientific witnesses... 369
Bad plumbing, dangers of... 74
Baltimore Medical and Surgical Society, proceedings of... 142
Bladder, rupture of... 152, 726
Bright's disease...46, 344
Benorrhœa, nitrate of silver for... 27
Board of Pharmaceutic Examiners, act establishing... 111
Boiled milk, injurious effects of... 736
Bromide of ethyl... 43
Buried at sea... 358
Blood vessels, physical examination of 403
Billwroth's operation... 488
Blindness, new remedy for... 304
Bell's paralysis... 166
Books, review of 52, 121, 183, 251, 307, ...375, 440, 503, 568, 631, 695, 758
Conjunctivitis catarrhalis acuta intermittens... 93
Carbolic acid poisoning... 48
Catalepsy... 94
Cannabis indica in migraine... 106
Carbuncles... 732
Cemeteries... 727
Convulsions, hysterical... 725
Chloroforms, death from... 744
Correspondence... 758
Capillary bronchitis... 663
Curious misuse of terms... 433
Chancroid... 427
Cholera infantum...474, 691
Croup... 529
Chlorosis... 90
Croupous pneumonia... 429
Chorea... 528
Chorea, arsenic in... 606
Cicatricial contractions... 665
Caconemia (milk-sickness)... 391
Clinical lecture... 36
Clinical thermometer...177, 402
Chloroform to sleeping persons... 367
Clinical reports... 521
Cundurango and carcinoma... 533
Chronic pelvic abscess... 106
Children's diseases, recent progress in treatment of... 108
Comparative mortality... 108
Cystitis, treatment of... 370
Code, breach of... [illegible]74
Corsets for scoliosis... 350
Consumption, treatment of... 245
Chancre, excision of... 176
Cerebral tumors in children... 234
Cyst of the pancreas... 303
Cough, why and how... 277
Carcinoma of the breast... 367
Carcinoma of vulva... 405
Carbolism... 25
Carbolic spasms... 26
Carbolic acid, death from... 27
Dengue in Columbus, Ga., in 1866... 675
Delay in first stage of natural labor... 449
Delirium tremens, convulsions of... 724
Diabetes mellitus... 726
Dermatology, practical notes on... 1
Diabetes mellitus, etc... 356
Diabetes and diseases of the uterus... 478
Diphtheria... 97
Diphtheria and tracheotomy... 24
Diphtheria, epidemic of... 264
Diphtheria, pilocarpin in... 24

Diphtheria, mycelium in...................... 94
Diphtheritic laryngitis, tracheotomy in .................................... 129
Diphtheritic paraplegia ....................... 346
Diseases of women, unnecessary operations in.................................... 45
Dissection..................................... 107
Diploma selling colleges...................... 47
Diplomas by medical colleges, act regulating..................................... 111
Dissection, veto of bill to legalize........ 118
Dihydroxyl benzoles......................... 229
Dislocated vertebræ.......................... 342
Dislocation of both lenses into the anterior chamber..................................... 5
Doctors in Norway............................ 619
Drainage and drainage tubes............... 92
Drainage and antiseptics..................... 294
Dyspepsia in nephritis........................ 480
Ear, effects of quinine on.................... 25
Ear, removal of foreign bodies from ... 243
Eczema, treatment of......................... 542
Eclampsia.......................................... 269
Eggs as food...................................... 620
Elegant pharmacy............................... 498
Ethics of New York............................ 432
Electricity in affections of joints ........ 533
Ether and chloroform......................... 172
Epidemic cerebro-spinal meningitis... 749
Erysipelas, treatment of..................... 741
Exophthalmic goitre, duboisine in...... 27
Explosive mixtures............................ 421
Eye, ear and throat clinic.................... 257
Eye and ear, diseases of, etc............... 231
False conception................................ 597
Faradism to palate.............................. 529
Faradization of neck, contraction of blood vessels in................................ 233
Female doctors in Philadelphia........... 567
Femoral aneurism, ligation for............ 641
Fistulæ and laparotomy...................... 266
Foreign bodies, removal of from ear... 243
Foreign substances in the body........... 579
Foreign body in the nose, orbit and cranium, etc...................................... 684
Food stuffs under the microscope......... 103
Fracture of ribs treated with plaster of Paris.......................................... 352
Fracture of the spinous processes of the cervical vertebræ...................... 723
Gastrotomy........................................ 304
Gastrotomy for intussusception........... 361
Gastritis in an infant, caused by mother's milk....................................... 472
Galvanism of tender regions............... 406
Genital disorders, etc......................... 292
General lower extremity splint............ 79
Glaucoma, operative measures in........ 135
Gratuitous medical services to the public.............................................. 371
Grape sugar and glucose..................... 609
Gonorrhœal rheumatism..................... 100
Gumma of iris................................... 271
Gunshot wound................................. 728
Gynæcology, recent progress in........... 289
Gynecic and obstetric practice, quinine in............................................. 280
Habit................................................ 621
Hæmatogenic albuminuria................. 91
Hahnemannism, otherwise called homœopathy...................................... 669
Hernia cured by the antiseptic use of animal ligature............................... 673
Hemorrhagic malarial fever................ 398
Hemorrhage...................................... 532
Hemorrhoids, treatment of................. 104
Heart diseases, treatment of............... 168
Headaches in children........................ 423
Herpes zoster..................................... 247
Hemorrhoids, external,....................... 365
Heart disease, some types of............... 193
Hiccough, pressure for........................ 349
Hip-joint diseases.............................. 733
Hysteria, cure of ............................... 95
Hermaphrodite, so-called.................... 356
Hospital report.................................. 477
Hot Springs of Arkansas..................... 513
How to cook rice................................ 753
Hydrocele, treatment of...................... 428
Hydrocele, curing without confinement............................................... 162
Hydrops articulorum intermittens...... 93
Hydrochinon..................................... 154
Hyperæmia of the brain, treatment of 479
Hyperidrosis of the feet...................... 298
Iodine in malaria................................ 433
Iodoform, topical use of...................... 546
Iodoform, bad effects from.................. 556
Iodoform, hypodermic injections of..... 229
Iodoform insanity............................... 497
Iodoform—its excellencies and its defects............................................... 604
Idioc salivation.................................. 577
Impure vaccine virus........................... 426
Imperforate anus, operation for........... 469
Intestinal obstruction, abdominal section for............................................. 413
Insanity, colony treatment for............. 600
Insanity and crime............................. 43
Insanity, bromide of potassium and quinine in........................................ 234
Infants' foods .................................... 475
International Medical Congress, proceedings of....................................... 12
International Medical Congress, section on obstetrics.............................. 82
Inoculation for pannus........................ 416
Intussusception.................................. 746
Intussusception, gastrotomy for.......... 361
Iron, preparations of suitable for hypodermic medication....................... 234
Is gonorrhœa specific?........................ 99
Items, 64, 126, 188, 254, 314, 381, 442, 506, ..........................................572, 634 699, 762
Jaborandi quebracho.......................... 304
Knife wound of the liver..................... 341
Labor, protoxide of nitrogen as an anæsthetic in.................................. 270
Labor, management of shoulders in... 241
Labor, use of nitrous oxide gas in........ 564
Law and prostitution.......................... 754
Lead colic......................................... 94
Leucocythemia.................................. 597
Leucorrhœa in children...................... 598
Leucorrhœa in children, treatment of 240
Localized muscular spasm.................. 231
Lithotomy......................................... 528
Liver, knife wound of........................ 341
Lively for surgeons............................ 176
Malignant growths............................ 304
Maternal impressions...................176, 402
Malarial fevers, chinoidine in............. 75
Maxilla, inferior, removal of.............. 27
Malaria, iodine in.............................. 433
Mastitis, strapping in......................... 45
Measels, the sequelæ of...................... 753
Medical organization......................... 305
Medical practice act........................... 116
Medical societies............................... 108
Medical expert testimony................... 181
Medical highways and byways............ 623
Medical student's primer.................... 431
Medical colleges and quackery...... 628, 757
Medical fees in London...................... 496
Medical societies, why not more prosperous?........................................ 559

Medical legislation in Georgia.......... 115
Medicine—a poem.......... 534
Melancholy, tinnitus the cause of.......... 353
Menstruation and its derangements... 272
Miliary tuberculosis simulating typhoid fever.......... 355
Milk indigestion in young children.. .. 171
Milk-sickness of upper Georgia (caconemia).......... 458
Milk-sickness (caconemia).......... 391
Milk, and the effect of drugs on.......... 42
Monstrosity.......... 401
Money question.......... 164
Methyl blue for coloring microscopic organisms.......... 93
Nasal polypi, treatment of.......... 174
Naso-pharyngeal catarrh, treatment of.......... 39
Naphthol..........26, 146
National jurisprudence.......... 424
Nitric acid and alcohol as a disinfectant.......... 94
Nephritis, chronic.......... 597
Nerve-stretching.......... 430
Nerve-stretching in ophthalmic-surgery.......... 232
Nephrectomy on account of uretero-uterine fistula.......... 91
Neuralgia, tuning fork for.......... 27
Neuralgia.......... 664
Neuralgia of the trigeminus.......... 532
New remedies.......... 49
New theories.......... 370
New York Pathological Society.......... 731
Nocturnal frights in children.......... 734
Nostrums, arrests for advertising.......... 494
Occlusion of vagina.......... 139
Occlusion of the gravid uterus.......... 240
Obstruction of the bowels, operation for.......... 396
Otology for general practitioners.......... 748
Oil of turpentine.......... 745
Oophorectomy..........481, 564
Ophthalmia neonatorum.......... 157
Ophthalmia in infants, prevention of. 532
Ovarian tumors, hints for the diagnosis of.......... 616
Ovaries, extirpation of.......... 28
Orange-colored pus.......... 268
Oxalate of cerium in vomiting and delirium tremens.......... 474
Pads, era of.......... 173
Palm, hemorrhage from, treatment of 688
Parallelism in the action of coniin and curare.......... 232
Pamphlets, 63, 125, 188, 254, 313, 380, 441, ..........505, 571, 633, 698, 761
Pancoast, the late Dr.......... 497
Pannus, inoculation for.......... 416
Paralysis, etc., from injuries to the head.......... 151
Paget, Sir James.......... 51
Pathological conditions in hanging..... 351
Perforation of the duodenum by ascarides.......... 407
Peppermint oil of, uses of.......... 622
Perineum, ruptures of, sutures in.......... 90
Pericardium, incision and drainage of 22
Peritoneal surgery, progress in.......... 155
Pneumonia followed by pleuritis.......... 662
Perineum, laceration of.......... 672
Perineum, rupture of, operation for..... 598
Pessary in vagina for six years.......... 263
Plumbing regulations.......... 552
Pressure in diseases of the uterus, ovaries and peri-uterine structures. 705
Phthisis, contagiousness of.......... 752
Phthisis laryngea, treatment of.......... 533
Physiology of the ox, etc.......... 539
Phimosis, dilatation in.......... 557
Popularizing medicine,.......... 248
Popliteal aneurism, case of.......... 300
Pneumonia, etiology of.......... 270
Pneumonia complicated with hiccough.......... 349
Pruritus ani.......... 368
Puerperal fever, intra-uterine treatment of.......... 268
Professional earnings in England.......... 558
Polypus of the ear, alcohol in treatment of.......... 23
Protoxide of nitrogen as an anæsthetic in labor.......... 270
Practice of medicine in Georgia, act regulating.......... 109
Prize essays.......... 180
Prize essays invited.......... 8
Prize essays and the Medical Association of Georgia.......... 694
Pressure in the external auditory meatus.......... 519
Procedentia uteri.......... 348
Prostatic obstruction, early treatment of.......... 484
Professional confidence.......... 500
Psoriasis.......... 480
Pompeian surgery.......... 680
Puerperal infection in the male.......... 427
Quackery in the profession.......... 407
Questionable surgery.......... 617
Quinine poisoning.......... 163
Quinine, indiscriminate use of.......... 170
Quinine, the use and abuse of.......... 250
Quinine, perfect solvent for.......... 302
Quinine in gynecic and obstetric practice.......... 280
Quinine and salycilic acid, action of on the ear.......... 406
Quinine, poisonous properties of.......... 163
Radius, fracture of from muscular contraction.......... 472
Rare tumors.......... 480
Rectal alimentation.......... 737
Rectum, ulcer of..........595, 612
Register, The.......... 49
Removing needles.......... 403
Resorcin.......... 150
Resorcin, what is it?.......... 364
Resorcin, uses of.......... 89
Retained placenta.......... 343
Retention of urine in elderly men...... 282
Retrospective.......... 756
Rheumatic nodosities.......... 91
Salicylic acid, is it a specific for rheumatism?.......... 496
Salt solution injected into the artery... 406
Sanitary condition of Bagshot Park... 560
Sanitary science.......... 107
Scrofulous glands.......... 688
Scurvy, etiology of.......... 352
Secale cornutum in varicose ulcers...... 93
Selecting spectacles.......... 608
Sewerage.......... 430, 614
Secondary suture of the ulner nerve... 533
Self-mutilation.......... 105
Sea-sickness, new remedy for.......... 11
Single breast in girls.......... 366
Skin eruptions following vaccination 480
Skin diseases, Chinese remedy for....... 153
Skin diseases, oleum gynocardiæ in.... 235
Small-pox and the public schools of Atlanta.......... 581
Small-pox in Georgia and its lesson..... 626
Small-pox and vaccination.......... 425
Small-pox, prevention and control of 321
Society rights.......... 502

Splinter, removal from eye.................. 397
Spinal curvature.................................... 410
Stammering............................................ 563
State licensing boards.......................... 418
State examinations in Alabama........... 494
Steno's duct, croupous inflammation of.........................................153, 232
Sterility in bovine twins......................... 368
Stomach, resection of........................... 271
Stigmata of maize and avena sativa... 10
Stomach, excision of............................. 44
Stomach, spontaneous rupture of........ 23
Suffocation from tobacco during chloroform narcosis.................................. 155
Surgical notes......................................... 32
Surgical uses of kangaroo tendon........ 493
Surgical millenium................................. 434
Surgical triumphs................................... 429
Surgery of the arteries.......................... 743
Sutures of nerves and tendons............ 228
Sutures of nerves, etc............................ 230
Symptoms simulating pregnancy........ 528
Syphilis.................................................... 529
Syphilis and irritation............................ 479
Syphilis, can a man have it twice ?..... 415
Syphilis, relation of to scrofula............ 486
Syphilis, early development of............ 724
Syphilis, action of mercury in.............. 169
Syphilis of the lungs.............................. 25
Syphilis, Cole's views of........................ 432
Syphilitic paraplegia.............................. 476
Tabes dorsalis, affections of joints in.............................................................. 354
Tea and coffee as nervines................... 740
Tetanus and trismus, etiology of......... 407
The American Medical Association and New York's new code................. 630
The blue color of water......................... 748
The New York code of ethics............... 501
The new anæsthetic............................... 48
The new ethics of the New York State Medical Society........................................ 437
The victims of the Ring theatre in Vienna..................................................... 490
The Medical Association of Georgia, proceedings of...................................... 525
The Medical Association of Georgia.... 435
Therapeutic nihilism.............................. 160
Thermcmeters, clinical, testing of........ 45
Thermometric bureau............................ 689
Thymol..................................................... 27
Tin poisoning........................................... 92
Too much originality and fortune....... 497
Tonsilitis, acute....................................... 682
Transitory albuminuria.......................... 405
Traumatic gangrene, amputation for.. 642
Tumors, treatment of by electrolysis... 404
Tumors of orbit........................................ 348
Trephining the skull, new method of.. 295
Tympanitic medical school.................... 175
Typhoid fever, bacteria in..................... 235
Typhoid fever, renal form of................. 355
Typhoid fever, bacillus of...................... 88
Ulcers of the lower extremity, the pathology and treatment of.......................... 205
Umbilical hernia..................................... 297
Umbilical hemorrhage, death from..... 495
Urine, extravasation of......................... 359
Uterus, action of pilocarpine on.......... 233
Urethral caruncle and cystitis.............. 685
Vaccination.............................177, 373, 385, 400
Vaccination and small-pox.................... 425
Vaccination in Atlanta............................ 646
Vaccination, skin eruption following 480
Vaccination with calf lymph................. 235
Valedictory............................................... 565
Variocele treated by excision of redundant scrotum...................................... 431
Vacillating physician.............................. 368
Venesection.............................................. 102
Venesection, therapeutics of................. 550
Vesical calculus, diagnosis of.............. 749
Vomiting, oxalate of cerium in............. 474
Wounds, antiseptic treatment of......... 735

